# Regulation of σ^B^-Dependent Biofilm Formation in *Staphylococcus aureus* through Strain-Specific Signaling Induced by Diosgenin

**DOI:** 10.3390/microorganisms11102376

**Published:** 2023-09-23

**Authors:** Seo-Young Kim, Minjun Kim, Tae-Jong Kim

**Affiliations:** Department of Forest Products and Biotechnology, Kookmin University, Seoul 02707, Republic of Korea

**Keywords:** biofilm, cell surface hydrophobicity, diosgenin, extracellular polysaccharide, extracellular protein, regulatory mechanism, *Staphylococcus aureus*

## Abstract

*Staphylococcus aureus* is a commensal skin bacterium and a causative agent of infectious diseases. Biofilm formation in *S. aureus* is a mechanism that facilitates the emergence of resistant strains. This study proposes a mechanism for the regulation of biofilm formation in *S*. *aureus* through strain-specific physiological changes induced by the plant steroid diosgenin. A comparison of diosgenin-induced changes in the expression of regulatory genes associated with physiological changes revealed the intracellular regulatory mechanisms involved in biofilm formation. Diosgenin reduced biofilm formation in *S*. *aureus* ATCC 6538 and methicillin-resistant *S*. *aureus* (MRSA) CCARM 3090 by 39% and 61%, respectively. Conversely, it increased biofilm formation in *S*. *aureus* ATCC 29213 and MRSA CCARM 3820 by 186% and 582%, respectively. Cell surface hydrophobicity and extracellular protein and carbohydrate contents changed in a strain-specific manner in response to biofilm formation. An assessment of the changes in gene expression associated with biofilm formation revealed that diosgenin treatment decreased the expression of *icaA* and *spa* and increased the expression of *RNAIII*, *agrA*, *sarA*, and *sigB* in *S*. *aureus* ATCC 6538 and MRSA CCARM 3090; however, contrasting gene expression changes were noted in *S*. *aureus* ATCC 29213 and MRSA CCARM 3820. These results suggest that a regulatory mechanism of biofilm formation is that activated *sigB* expression sequentially increases the expression of *sarA*, *agrA*, and *RNAIII*. This increased *RNAIII* expression decreases the expression of *spa*, a surface-associated adhesion factor. An additional regulatory mechanism of biofilm formation is that activated *sigB* expression decreases the expression of an unknown regulator that increases the expression of *icaA*. This in turn decreases the expression of *icaA*, which decreases the synthesis of polysaccharide intercellular adhesins and ultimately inhibits biofilm formation. By assessing strain-specific contrasting regulatory signals induced by diosgenin in *S*. *aureus* without gene mutation, this study elucidated the signal transduction mechanisms that regulate biofilm formation based on physiological and gene expression changes.

## 1. Introduction

Biofilms facilitate the survival of microorganisms by interfering with the penetration of antibiotics [1,2] and the action of the host’s immune cells [3,4]. More than 65% of microbial infections are related to biofilm formation [5]. Biofilms do not act as a barrier to completely block antibiotic penetration [6]; instead, they reduce the antibiotic concentration to below the effective bactericidal concentration [7]. In addition, cells in biofilms receive limited nutrients; therefore, their growth rate decreases and their antibiotic sensitivity becomes lower than that of planktonic cells [8]. During the treatment of biofilm-related bacterial infections, continuous antibiotic prophylaxis is followed because of low antibiotic susceptibility, resulting in the emergence of antibiotic-resistant strains [9]. Cells in a biofilm cannot be easily removed by antibiotics and can also cause chronic infections [10]. Therefore, inhibiting biofilm formation has been proposed as a way to control bacterial infections [11,12].

During the discovery of biofilms, polysaccharides likely accounted for most of the materials surrounding biofilm cells; therefore, they were simply labeled as slime or glycocalyx, suggesting that they are sticky and have adhesive properties [13]. However, further research revealed the presence of polysaccharides, proteins, environmental DNA, and lipids in biofilms; therefore, they were labeled as extracellular polymeric substances (EPS) [14]. During biofilm formation, cell surface hydrophobicity [15], flagella [16,17], environmental RNA [18], and EPS [19] are known as factors affecting initial adhesion. Studies have also demonstrated that a decrease in cell surface hydrophobicity is one of the main causes of reduced biofilm formation [13,20,21].

*Staphylococcus aureus* is a Gram-positive commensal and opportunistic pathogen. This bacterium can cause many infections and diseases, including skin infections [22,23], endocarditis [24], sepsis [25], toxic shock syndrome [26], and medical device-associated infections [27]. Planktonic cells are associated with acute infections, such as bacteremia and skin abscesses [28]. Cells in biofilms can attach to host tissues, such as bones or heart valves, and cause chronic infections [29]. Many methicillin-resistant *S. aureus* (MRSA) strains are multidrug-resistant strains that are resistant to beta-lactam antibiotics and other antibiotics [30]. The biofilm-forming ability of MRSA aggravates the risk of severe infectious diseases [31] and increases mortality [32].

Diverse intracellular regulatory mechanisms have been proposed for biofilm formation in *S*. *aureus*. The regulatory mechanisms involved in the formation of EPS are direct cellular mechanisms that determine biofilm formation. The polysaccharide intercellular adhesin (PIA) is a well-known substance responsible for biofilm formation; it facilitates adhesion and aggregation during biofilm formation [33,34]. PIA is composed of poly-β(1-6)-N-acetylglucosamine and is synthesized by membrane proteins encoded by the *icaADBC* operon [35]. Surface-related adhesins (SRAs) are also crucial factors associated with biofilm formation in *S*. *aureus*, particularly in strains lacking PIA [36,37]. Spa is a well-known SRA that is involved in both biofilm formation and host cell binding [37,38].

Intercellular signaling mechanisms play a crucial role in biofilm formation. One such mechanism involves the *agr* system, which is a part of the quorum-sensing system [39]. The *agr* system is considered to inhibit biofilm formation by interfering with the expression of SRAs through the activation of *RNAIII* expression [39]. Moreover, SarA, a global regulator of *S*. *aureus* virulence factors, plays an important role in biofilm formation [34]. The expression of *sarA* is activated by σ^B^, an alternative general stress response sigma factor [40]. Increased *sarA* expression has been found to inhibit biofilm formation by activating the *agr* system [40]. These results suggest a complex interplay between the *agr* system, SarA, and σ^B^ in regulating biofilm formation in *S*. *aureus*.

Diosgenin is a compound that accounts for most of the saponins present in fenugreek and hemp; it is an important precursor widely used in the pharmaceutical industry for the synthesis of oral contraceptives, sex hormones, and other steroids [41]. It can be absorbed through the intestine and participate in the regulation of cholesterol metabolism [42]. In addition, it can exhibit anti-inflammatory effects by inhibiting the production of enzymes [43] and anti-cancer effects by promoting the production of p53, a cancer-suppressing protein [44]. It also exhibits antibacterial activity against planktonic and biofilm cells of the bacteria *Porphyromonas gingivalis* and *Prevotella intermedia*; however, the underlying mechanism has not been elucidated [45]. Conversely, diosgenin exhibits a low antibacterial activity against some *S*. *aureus* strains in the planktonic state [46]. The effects of diosgenin on *S*. *aureus* biofilms remain unknown. However, tea saponin can inhibit biofilm formation in *Streptococcus agalactiae* [47], and the chemical derivatives of *Camellia oleifera* sapogenin can effectively inhibit biofilm formation in *S*. *aureus* and *Escherichia coli*. Furthermore, sapogenin derivatives may target mannitol-1-phosphate dehydrogenase to inhibit biofilm formation via a molecular docking method [48].

During our research on compounds that inhibit biofilm formation in *S*. *aureus*, we discovered that diosgenin could impact biofilm formation. Interestingly, the effect of diosgenin on biofilm formation differed depending on the specific *S*. *aureus* strain. In some strains, diosgenin had no significant effect on biofilm formation, while in others, it either increased or decreased biofilm formation. These strain-specific changes in biofilm formation could help trace signal transduction pathways for identifying the physiological regulatory mechanisms of biofilm formation in cells without gene alterations.

The regulatory mechanisms of biofilm formation in *S*. *aureus* have been extensively investigated. This accumulation of knowledge has led to the development of various models that aim to unravel the complex processes underlying biofilm formation in *S*. *aureus*. This study will verify the biofilm formation regulation model proposed in many previous studies through mutation of genes and propose an improved model using a new method, mutation-free signaling of external environmental changes. We also highlighted strain-specific responses, including contrasting responses related to the activity of σ^B^.

## 2. Materials and Methods

### 2.1. Strains and Culture Medium

*S. aureus* ATCC 6538 was purchased from the Korean Collection for Type Cultures at the Korea Research Institute of Bioscience and Biotechnology (Jeongeup, Republic of Korea). *S*. *aureus* ATCC 29213 was obtained from the American Type Culture Collection (Manassas, VA, USA). MRSA CCARM 3090, MRSA CCARM 3806, MRSA CCARM 3820, MRSA CCARM 3846, MRSA CCARM 3862, MRSA CCARM 3876, MRSA CCARM 3878, MRSA CCARM 3879, and MRSA CCARM 3905 were purchased from the Culture Collection of Antimicrobial Resistant Microbes (Korea National Research Resource Center, Seoul Women’s University, Seoul, Republic of Korea). Eight *S*. *aureus* strains (CN-OA1, CN-OA2, FH-OA6, JN-OA2, KN-OA2, AP-OA1, NE-1A1, and CK-OA1) were obtained from the bacterial strain collection of the BioResource laboratory in Kookmin University (Seoul, Republic of Korea). All bacteria were mixed with 25% glycerol and stored at −80 °C.

Bacteria stored at −80 °C were streaked onto tryptic soy agar (TSA, ref: 214010, Becton, Dickinson and Company Korea Ltd., Seoul, Republic of Korea) and incubated at 37 °C for 24 h. A single colony was inoculated into 5 mL of tryptic soy broth (TSB, ref: 211825, Becton, Dickinson, and Company Korea Ltd.) and cultured at 37 °C for 24 h at 250 rpm to obtain precultured cells.

### 2.2. Cell Growth Curve

Growth curves were observed for 24 h to determine whether diosgenin (catalog number: sc-205652, Santa Cruz Biotechnology Inc., Dallas, TX, USA) affected the growth of *S*. *aureus*. Diosgenin was dissolved in ethanol to overcome its solubility in water and added at a concentration of 80 µM, which significantly affected biofilm formation, to 20 mL TSB containing 0.5% glucose in a 250 mL baffled flask. Precultured *S*. *aureus* was subcultured into the main culture medium to obtain 2.0 × 10^7^ colony-forming units (CFU)/mL and then cultured at 37 °C at 250 rpm for 24 h. Cell density was measured based on absorbance at 600 nm (Abs_600_).

### 2.3. Evaluation of Biofilm Formation

A biofilm formation experiment was conducted using a 96-well polyvinyl chloride (PVC) microplate (catalog number: 2797, Corning Korea Company Ltd., Seoul, Republic of Korea). TSB containing 0.5% glucose and 80 µM diosgenin was dispensed into each well of the 96-well PVC plate at a total volume of 100 µL. The precultured bacteria were subcultured to obtain a final inoculation concentration of 2.0 × 10^7^ CFU/mL. The cells were incubated at 37 °C for 24 h.

The degree of biofilm formation was measured using 1% crystal violet according to previously described methods [17], with some modifications. Suspended cells were removed from the cultured 96-well PVC microplate and washed thrice with distilled water. In total, 100 µL of 1% crystal violet was aliquoted and removed after the culture was allowed to stand at 23 °C for 15 min. The wells were again washed thrice with distilled water. Following this, 100 µL of 95% ethanol was dispensed on the dyed biofilm and incubated at 23 °C for 15 min. The biofilm was quantitatively evaluated by measuring Abs_600_ using Synergy™ LX Multi-Mode Reader (BioTek Instruments Korea Ltd., Seoul, Republic of Korea).

### 2.4. Measurement of Cell Hydrophobicity

The effect of diosgenin (80 µM) on bacterial cell surface hydrophobicity was also evaluated. For this purpose, cell surface hydrophobicity was examined according to previously described methods [49], with some modifications. In brief, 4 mL TSB containing 0.5% glucose and 80 µM diosgenin was added into a test tube. The precultured bacteria were then subcultured to 2.0 × 10^7^ CFU/mL. After incubation at 37 °C at 250 rpm for 18 h, the cells were harvested by centrifugation (4300 ×*g* for 10 min), washed twice with phosphate-buffered saline (PBS, catalog number: P5493, Sigma-Aldrich Co., St. Louis, MO, USA), and resuspended in 4 mL PBS to measure Abs_600_ (A_0_). Then, 0.4 mL *n*-hexadecane was added, mixed well by vortexing for 1 min, and incubated at 23 °C for 15 min.

Abs_600_ (A) of the lower aqueous layer out of the two separated layers was measured. Hydrophobicity was calculated using the following formula:

Hydrophobicity (%) = [(A_0_ − A)/A_0_] × 100 (1)

### 2.5. Analysis of Gene Expression Levels Using Real-Time Polymerase Chain Reaction (RT-PCR)

The expression levels of biofilm-related genes were analyzed using RT-PCR to determine the effect of 80 µM diosgenin on gene expression. In brief, 5 mL of TSB with 0.5% glucose and 80 µM diosgenin was added into a test tube. The precultured bacteria were then subcultured to 2.0 × 10^7^ CFU/mL. After incubation at 37 °C with shaking at 250 rpm for 1 h, the total RNA was extracted using the AccuPrep^®^ Bacterial RNA Extraction Kit (Bioneer Co., Daejeon, Republic of Korea), according to the manufacturer’s instructions. As *S*. *aureus* is a Gram-positive bacterium, TissueLyser LT (Qiagen, Seoul, Republic of Korea) was used to break the cell wall at 50 Hz for 5 min.

The biofilm-related genes *agrA*, *icaA*, *RNAIII*, *sarA*, *sigB*, and *spa* were selected, and changes in their expression levels induced by 80 µM diosgenin were analyzed. The primer sequences for amplification were 5′-CCACACTGGAACTGAGACAC-3′ and 5′-AAGACCTTCATCACTCACGC-3′ for 16S rRNA, 5′-GCTTTGTCGTCAATCGCCAT-3′ and 5′-TCACCGATGCATAGCAGTGT-3′ for *agrA*, 5′-TGAACCGCTTGCCATGTG-3′ and 5′-CACGCGTTGCTTCCAAAGA-3′ for *icaA*, 5′-TTCACTGTGTCGATAATCCA-3′ and 5′-GGAAGGAGTGATTTCAATGG-3′ for *RNAIII* [50], 5′-TCTCTTTGTTTTCGCTGATGT-3′ and 5′-TCAATGGTCACTTATGCTGACA-3′ for *sarA*, 5′-GCGGTTAGTTCATCGCTCAC-3′ and 5′-AGTGTACATGTTCCGAGACGT-3′ for *sigB*, and 5′-TGTTGTCTTCCTCTTTTGGTGC-3′ and 5′-AGACGATCCTTCAGTGAGCA-3′ for *spa*. Gene expression levels were analyzed using the AccuPower^®^ RT Premix (Bioneer Co.) for cDNA synthesis and PowerUp™ SYBR™ Green Master Mix (ThermoFisher Scientific Korea Ltd., Seoul, Republic of Korea) for RT-PCR, according to the manufacturers’ instructions. RT-PCR analysis of all genes was performed under the following conditions: UDG activation (50 °C, 2 min); Dual-Lock™ DNA polymerase activation (95 °C, 2 min); polymerization (40 cycles of denaturation [95 °C, 15 s], annealing [57 °C, 15 s for 16S rRNA, *agrA*, *icaA*, *sarA*, *sigB*, and *spa* and 53 °C, 15 s for *RNAIII*], and elongation [72 °C, 1 min]); and melting (denaturation [95 °C, 15 s], annealing [60 °C, 1 min], and dissociation [95 °C, 1 s]). RT-PCR was performed using QuantStudio5 (ThermoFisher Scientific Korea Ltd.). Cycle threshold (Ct) values for each gene were obtained and standardized using 16S rRNA, a housekeeping gene. Gene expression levels were compared using the 2^−ΔΔCt^ method.

### 2.6. Quantitative Analysis of EPS

EPS was analyzed according to previously described methods [51], with some modifications. In brief, 5 mL TSB with 0.5% glucose and 80 µM diosgenin was added into a test tube. The precultured bacteria were then subcultured to 2.0 × 10^7^ CFU/mL. After incubation at 37 °C with shaking at 250 rpm for 1, 6, or 18 h, the cells were harvested by centrifugation (4300× *g* for 10 min). The cell-free supernatant was collected and stored at −80 °C. After the cell pellet was washed once with PBS, 5 mL of isotonic buffer (10 mM Tris/HCl at pH 8.0, 10 mM EDTA, and 2.5% NaCl) was added. The pellet was then incubated at 4 °C for 12 h. After 3 min of vigorous mixing, another cell-free supernatant was obtained by centrifugation (4300× *g* for 10 min). After the two cell-free supernatants were mixed, three times the volume of ice-cold ethanol was added to the mixture. The mixture was then incubated at −20 °C for 12 h. The supernatant was again removed by centrifugation (4300× *g* for 10 min). Following this, the precipitated pellet was dried at 23 °C. Dried EPS was obtained and dissolved in distilled water for analysis.

The proteins in the EPS were quantitatively analyzed using the Bradford method [52]. In brief, 100 µL of dissolved EPS was completely mixed with 1 mL of Bradford reagent (Biosesang, Seongnam, Republic of Korea) and incubated at 23 °C for 2 min. Following this, the absorbance at a wavelength of 595 nm was measured.

The polysaccharides in the dried EPS were quantitatively analyzed using phenol–sulfuric acid according to a previously described procedure [53], with some modifications. In brief, 200 µL of dissolved EPS and 600 µL of sulfuric acid were mixed vigorously. Following this, 120 µL of 5% phenol was added and incubated at 23 °C for 10 min. The absorbance at a wavelength of 490 nm was then measured.

### 2.7. Statistical Analysis

Statistical significance was assessed using a *t*-test. Data were statistically analyzed by comparing the values of the control group with those of the experimental group.

## 3. Results and Discussion

### 3.1. Strain-Specific Effects of Diosgenin on Biofilm Formation

**Figure 1:**
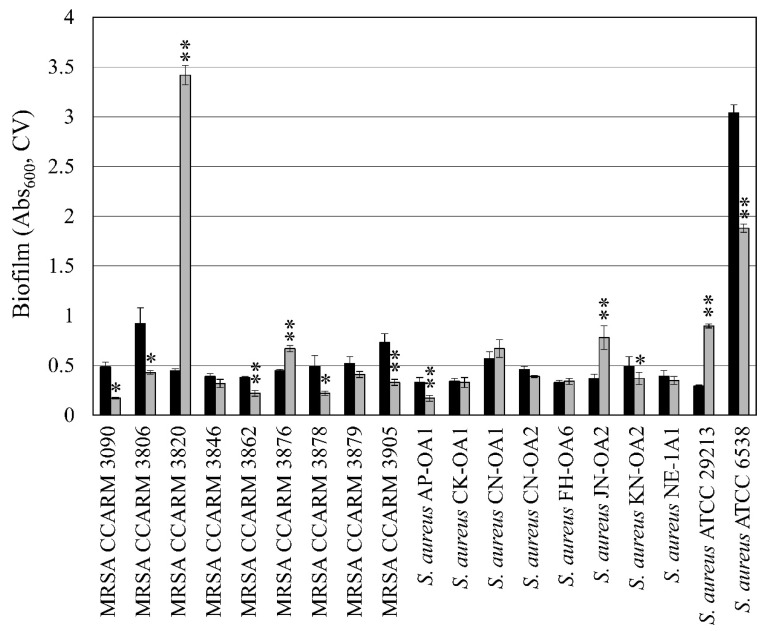
Changes in biofilm formation in *Staphylococcus aureus* strains after diosgenin treatment for 24 h. Black bars represent the amount of biofilm formed in the absence of diosgenin as a control. Gray bars represent the amount of biofilm formed with 80 μM diosgenin. The amount of biofilm was measured using crystal violet (CV). Values were calculated from five independent experiments, and their standard deviations are shown. Values that differ from the control with 95% and 99% confidence levels are marked with one and two asterisks, respectively, on top of the bars.

In our screening experiments, diosgenin was identified as affecting biofilm formation in *S*. *aureus*. To determine whether the effects of diosgenin on biofilm formation are common to all *S*. *aureus* species, diosgenin-induced changes in biofilm formation were evaluated in 19 different *S*. *aureus* strains, including 9 MRSA strains (Figure 1). Of the 19 tested strains, no diosgenin-induced change in biofilm formation was noted in 7 strains. However, biofilm formation increased in four strains and was inhibited in eight strains. These results suggested that the effect of diosgenin on biofilm formation was strain-specific. To elucidate the physiological changes and intracellular signaling mechanisms by which diosgenin influences biofilm formation, we selected *S*. *aureus* ATCC 6538 and *S*. *aureus* ATCC 29213, which have reported whole genome sequences. These strains showed contrasting changes in biofilm formation in response to diosgenin. Among the remaining tested strains, we selected MRSA CCARM 3090 and MRSA CCARM 3820, which showed the greatest decrease and increase, respectively, in biofilm formation in response to diosgenin.

### 3.2. Effect of Diosgenin on Cell Growth and Biofilm Formation

**Figure 2:**
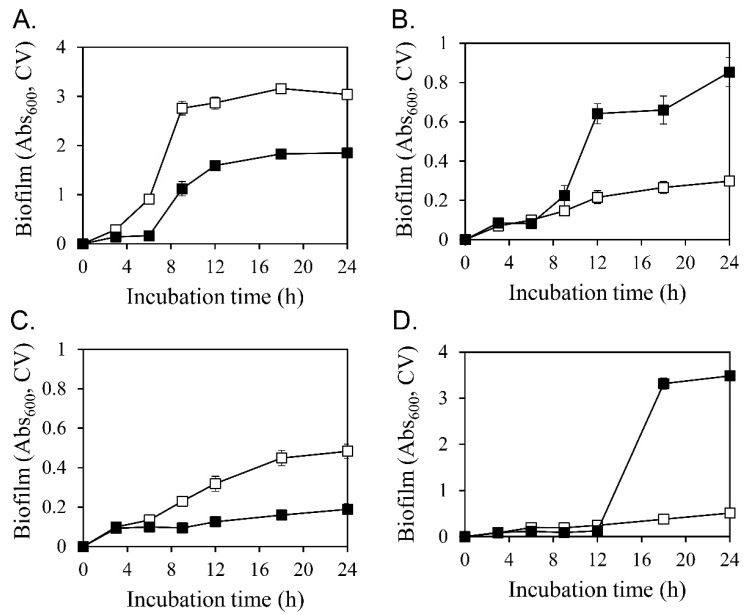
Effect of diosgenin on biofilm formation. Biofilm formation in *Staphylococcus aureus* ATCC 6538 (**A**), *S. aureus* ATCC 29213 (**B**), MRSA CCARM 3090 (**C**), and MRSA CCARM 3820 (**D**) was determined using crystal violet (CV) without diosgenin (☐, control) or with 80 μM diosgenin (■). Values were calculated from five independent results, and their standard deviations are shown.

The four selected strains were treated with 80 μM diosgenin, and changes in biofilm formation (Figure 2) and cell growth (Supplementary Figure S1) were observed. Diosgenin inhibited biofilm formation in *S*. *aureus* ATCC 6538 and MRSA CCARM 3090 by 39% and 61%, respectively. Conversely, it increased biofilm formation in *S*. *aureus* ATCC 29213 and MRSA CCARM 3820 by 186% and 582%, respectively. These findings confirmed that diosgenin significantly changed biofilm formation in S. *aureus*, resulting in two contrasting types of responses.

Biofilm formation is closely related to changes in bacterial growth. After treatment with 80 µM diosgenin, the growth of four strains, namely *S*. *aureus* ATCC 6538, *S*. *aureus* ATCC 29213, MRSA CCARM 3090, and MRSA CCARM 3820, was measured to determine the effect of diosgenin on cell growth (Supplementary Figure S1). In all of the tested strains, growth was slightly delayed by 80 μM diosgenin; however, the difference was not significant.

The minimum inhibitory concentration (MIC) was measured to determine the bactericidal activity of diosgenin against the four *S*. *aureus* strains; however, growth inhibition was not observed at the maximum soluble concentration (160 μM) in water-based media. Although the antibiotic activity of diosgenin has not been well described, its antibacterial activity against *P. gingivalis* and *P. intermedia* has been reported [45]. The MIC for *S*. *aureus* ATCC 25923 was 0.98 mM. Moreover, in MRSA 10, no growth inhibition was noted at the maximum concentration of 2.47 mM [54].

### 3.3. Diosgenin-Induced Changes in Cell Surface Hydrophobicity

**Figure 3:**
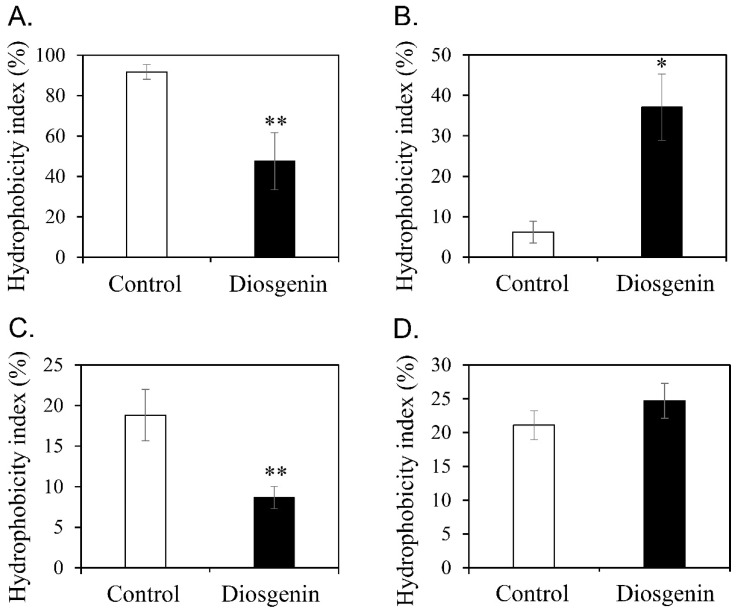
Diosgenin-induced changes in cell surface hydrophobicity. The cell surface hydrophobicity of *Staphylococcus aureus* ATCC 6538 (**A**), *S. aureus* ATCC 29213 (**B**), MRSA CCARM 3090 (**C**), and MRSA CCARM 3820 (**D**) was examined without diosgenin (open bars, control) or with 80 μM diosgenin (closed bars). Values were calculated from four independent results, and their standard deviations are shown. Values that differ from the control with 95% and 99% confidence levels are marked with one and two asterisks, respectively, on top of the bars.

Biofilm formation was evaluated in a 96-well PVC microplate (Figure 2). The PVC surface is hydrophobic. It is advantageous for microbial cells to be hydrophobic to initiate biofilm formation. Diosgenin-induced changes in the cell surface hydrophobicity of *S*. *aureus* were assessed (Figure 3). After treatment with 80 µM diosgenin, the cell surface hydrophobicity indices of *S*. *aureus* ATCC 6538 and MRSA CCARM 3090 significantly decreased (Figure 3A,C). Conversely, the cell surface hydrophobicity index of *S*. *aureus* ATCC 29213 significantly increased (Figure 3B). Furthermore, the average cell surface hydrophobicity index of MRSA CCARM 3820 increased, albeit not significantly (Figure 3D). The surface hydrophobicity of a solid object and that of cells must match to allow bacteria to sufficiently form a biofilm on the surface of a solid object. The surface hydrophobicity of the PVC plate in this study could support biofilm formation by cells with a hydrophobic surface. The diosgenin-induced decrease in the hydrophobicity indices of *S*. *aureus* ATCC 6538 and MRSA CCARM 3090 was associated with the reduced attachment of cells to the PVC surface during biofilm formation. Conversely, the diosgenin-induced increase in the hydrophobicity indices of *S*. *aureus* ATCC 29213 and MRSA CCARM 3820 was associated with increased cell attachment. As shown in Figure 3, the diosgenin-induced changes in cell surface hydrophobicity were consistent with the expected changes in biofilm formation on the hydrophobic PVC surface.

A previous study has shown that biofilm formation changes are proportional to variations in the cell surface hydrophobicity of *S*. *aureus* depending on pH [55]. Temperature-dependent changes in cell surface hydrophobicity were found to be associated with biofilm formation in 67 *S*. *aureus* isolates [49]. Vitexin, an apigenin flavone glucoside, could reduce *S*. *aureus* cell surface hydrophobicity and biofilm formation at the sub-MIC [56]. These results suggest that diosgenin-induced changes in cell surface hydrophobicity are a major cause of alterations in biofilm formation.

### 3.4. Diosgenin-Induced Changes in Extracellular Protein Contents

**Figure 4:**
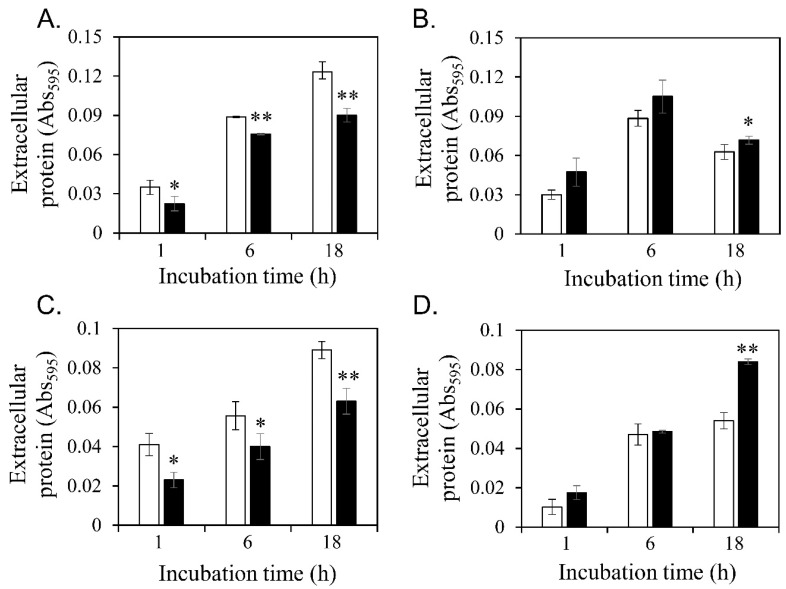
Diosgenin-induced changes in extracellular protein contents. The extracellular protein contents of *Staphylococcus aureus* ATCC 6538 (**A**), *S. aureus* ATCC 29213 (**B**), MRSA CCARM 3090 (**C**), and MRSA CCARM 3820 (**D**) were measured without diosgenin (open bars, control) or with 80 μM diosgenin (closed bars). Values were calculated from two independent results, and their standard deviations are shown. Values that differ from the control with 95% and 99% confidence levels are marked with one and two asterisks, respectively, on top of the bars.

Extracellular protein contents are closely correlated with biofilm formation [57,58]. The changes induced by 80 μM diosgenin in the extracellular protein contents of planktonic cells were quantitatively measured (Figure 4). After 1, 6, and 18 h of diosgenin treatment, the extracellular protein contents of *S*. *aureus* ATCC 6538 and MRSA CCARM 3090 were significantly lower than those of untreated cells (Figure 4A,C). The extracellular protein contents of *S*. *aureus* ATCC 29213 and MRSA CCARM 3820 were increased by diosgenin treatment at the mean value; however, statistically significant differences with 95% were observed only at 18 h (Figure 4B,D). Considering the correlation between extracellular protein contents and biofilm formation, the diosgenin-induced changes in the extracellular protein contents of planktonic cells could affect biofilm formation in *S*. *aureus*. In addition, such changes may induce variations in the degree of cell surface hydrophobicity. The extracellular protein content shown in Figure 4 was correlated with biofilm formation. A previous study suggested the importance of proteins in biofilm formation through the inhibition of biofilm formation by protease treatment [59].

### 3.5. Diosgenin-Induced Changes in Extracellular Polysaccharide Contents

**Figure 5:**
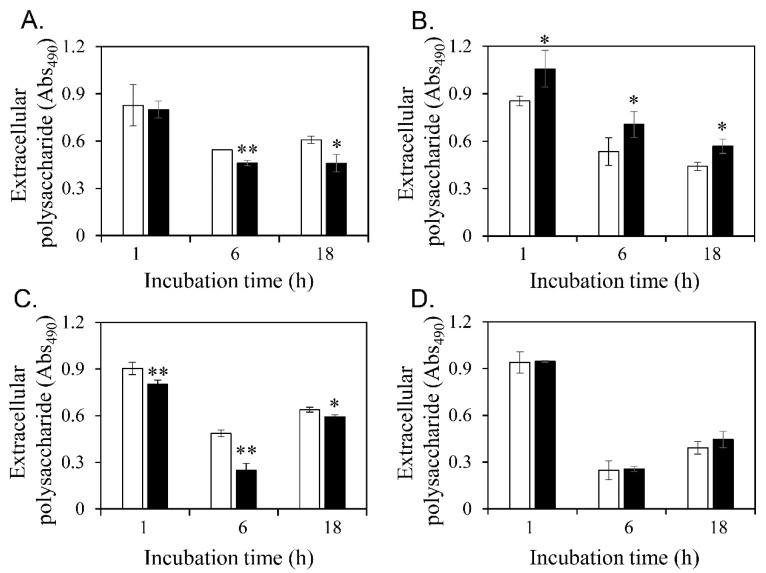
Diosgenin-induced changes in extracellular polysaccharide contents. The extracellular polysaccharide contents of *Staphylococcus aureus* ATCC 6538 (**A**), *S. aureus* ATCC 29213 (**B**), MRSA CCARM 3090 (**C**), and MRSA CCARM 3820 (**D**) were measured without diosgenin (open bars, control) or with 80 μM diosgenin (closed bars). Values were calculated from two independent results, and their standard deviations are shown. Values that differ from the control with 95% and 99% confidence levels are marked with one and two asterisks, respectively, on top of the bars.

Extracellular polysaccharides are materials that form biofilms and are closely related to biofilm formation [60,61]. Diosgenin-induced changes in the extracellular polysaccharide contents of planktonic cells were measured (Figure 5). The extracellular polysaccharide contents of *S*. *aureus* ATCC 6538 and MRSA CCARM 3090 decreased 6 and 18 h after diosgenin treatment (Figure 5A,C). Conversely, the extracellular polysaccharide contents of *S*. *aureus* ATCC 29213 increased 1, 6, and 18 h after diosgenin treatment (Figure 5B). The average extracellular polysaccharide contents of MRSA CCARM 3820 slightly increased, albeit not significantly (Figure 5D).

### 3.6. Diosgenin-Induced Changes in the Expression of Genes Associated with the Regulation of Biofilm Formation

**Figure 6:**
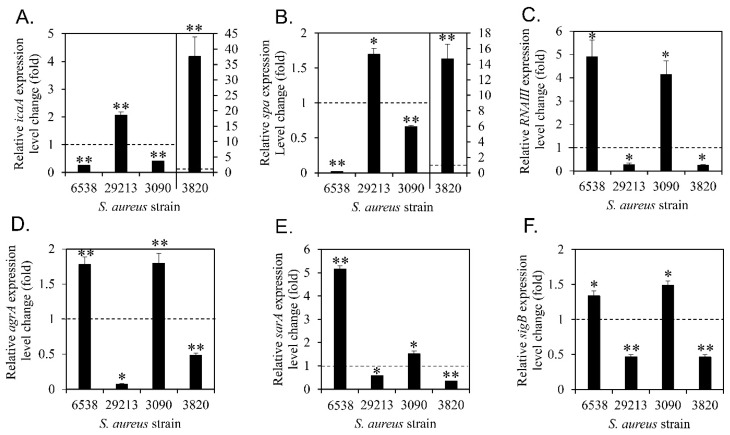
Diosgenin-induced changes in gene expression levels. Gene expression changes in *icaA* (**A**), *spa* (**B**), *RNAIII* (**C**), *agrA* (**D**), *sarA* (**E**), and *sigB* (**F**) were calculated by comparing the gene expression levels with 80 μM diosgenin treatment for 1 h with those without diosgenin treatment using RT-PCR. The dotted horizontal line indicates no change in gene expression levels induced by diosgenin with a value of 1. Values were calculated from three independent results, and their standard deviations are shown. Values that differ from the control with 95% and 99% confidence levels are marked with one and two asterisks, respectively, on top of the bars.

Diosgenin changed the extracellular protein (Figure 4) and polysaccharide (Figure 5) contents; these changes were proportional to the change in biofilm formation. Diosgenin-induced changes in the expression of genes involved in the regulation of physiological changes related to biofilm formation were measured (Figure 6). Gene expression changes were observed during planktonic growth.

PIA is a well-known extracellular polysaccharide associated with biofilm formation in *S*. *aureus*. It is synthesized by proteins expressed at the *ica* locus [33]. In this study, diosgenin-induced changes in the expression of *icaA*, the first gene in the *icaADCB* operon, were measured (Figure 6A). Diosgenin treatment decreased *icaA* expression in *S*. *aureus* ATCC 6538 and MRSA CCARM 3090, in which biofilm formation and extracellular polysaccharide contents were reduced. Conversely, diosgenin treatment increased *icaA* expression in *S*. *aureus* ATCC 29213 and MRSA CCARM 3820, in which biofilm formation and extracellular polysaccharide contents were increased. Changes in the extracellular polysaccharide contents and *icaA* expression along with the changes in biofilm formation induced by diosgenin suggested that extracellular polysaccharides, such as PIA, were a cause of diosgenin-induced changes in biofilm formation.

Among the proteins involved in biofilm formation, Spa promotes cell aggregation [61]. Diosgenin-induced changes in *spa* expression were also measured (Figure 6B). Diosgenin treatment decreased *spa* expression in *S*. *aureus* ATCC 6538 and MRSA CCARM 3090. Conversely, it increased *spa* expression in *S*. *aureus* ATCC 29213 and MRSA CCARM 3820. These findings indicated that *spa* expression was proportional to biofilm formation and extracellular protein contents.

An *S*. *aureus* mutant in which the *spa* function was lost could no longer form a biofilm. When the Spa protein was added to the culture medium of this *spa* mutant, its ability to form a biofilm was restored [61]. Spa is not covalently attached to cells and is implicated in biofilm formation. It can bind to IgG antibodies that inhibit the attachment of microorganisms to a silastic catheter and interfere with its activity [62].

Increased *RNAⅢ* expression inhibits *spa* expression and decreases biofilm formation [63]. RNAⅢ is an effector of the quorum-sensing system; it regulates transcriptional regulators and virulence factors [64]. AgrA activates *RNAⅢ* expression [65]. The *agr* system is a two-component regulatory system that regulates many virulence factors in *S*. *aureus* and plays an important role in the early stages of biofilm formation [64,66]. SarA is a global regulator that controls *agrA* expression. SarA expression is known to be regulated by σ^B^, which is an alternative sigma factor that regulates the expression of many genes in response to external environmental stresses.

In this study, diosgenin-induced changes in the expression of *RNAⅢ*, *agrA*, *sarA*, and *sigB* were measured (Figure 6C,F). The expression levels of the four aforementioned genes were increased by diosgenin treatment in *S*. *aureus* ATCC 6538 and MRSA CCARM 3090 and decreased in *S*. *aureus* ATCC 29213 and MRSA CCARM 3820. These results suggested that diosgenin could regulate the expression of proteins related to biofilm formation in a strain-specific manner.

### 3.7. Regulatory Signaling Mechanisms of Biofilm Formation Identified Using Diosgenin

**Figure 7:**
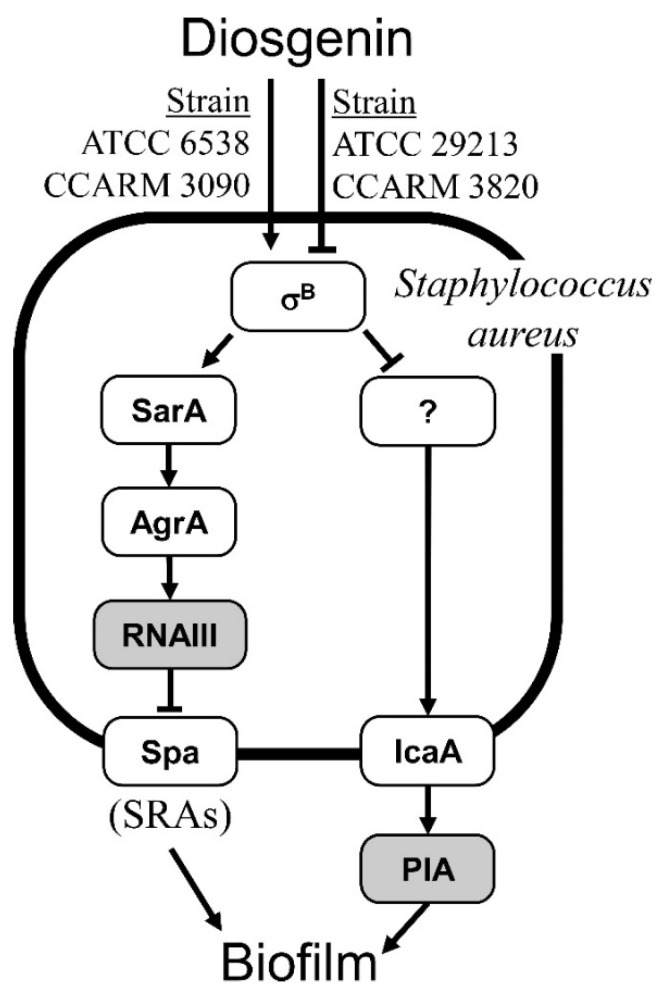
A regulatory signaling model of biofilm formation. Diosgenin activated σ^B^ expression in *Staphylococcus aureus* ATCC 6538 and MRSA CCARM 3090 and inhibited σ^B^ expression in *S*. *aureus* ATCC 29213 and MRSA CCARM 3820. In the signal transduction pathway on the left side of the figure, σ^B^ sequentially promotes the expression of SarA and AgrA, which in turn promotes RNAIII expression. Increased RNAIII expression inhibits the expression of surface-related adhesins (SRAs), increasing biofilm formation. The other signal transduction pathway, shown on the right side of the figure, is regulated by the σ^B^-mediated inhibition of an unknown regulator that promotes the expression of IcaA, which synthesizes the polysaccharide intercellular adhesin (PIA), increasing biofilm formation. In conclusion, σ^B^, an alternative general stress response sigma factor, inhibits biofilm formation.

The proposed regulatory mechanism of biofilm formation using diosgenin is shown in Figure 7. Diosgenin activates or represses *sigB* expression in a strain-specific manner. σ^B^ activates *RNAIII* expression by sequentially increasing *sarA* and *agrA* expression. RNAIII inhibits biofilm formation by repressing *spa* expression. In this study, *spa* expression was correlated with biofilm formation after diosgenin treatment, as opposed to *sigB* expression. The changes in the strain-specific gene expression after diosgenin treatment (Figure 6) along with the changes in biofilm formation support the regulatory model of biofilm formation proposed in Figure 7.

An additional regulatory mechanism of biofilm formation is that σ^B^ increases the turnover of Ica proteins and eventually reduces the synthesis of PIA [67]. In our gene expression observations, the change in *sigB* expression induced by diosgenin treatment was opposite to the change in *icaA* expression. This suggests that in addition to the increased turnover rate of IcaA by σ^B^, diosgenin treatment may be involved in the expression of *icaA*. A proposed mechanism is that σ^B^ represses *icaA* expression by inhibiting the expression of unknown genes that activate *icaA* expression [68]. The gene expression patterns noted in response to diosgenin treatment in this study support the hypothesis that σ^B^ is involved in *icaA* expression. However, future research should clarify whether *icaA* expression changes are regulated by σ^B^ and identify the intermediate gene between σ^B^ and *icaA* in the signal transduction pathway.

A previous study has suggested that SarA promotes *icaA* expression [34]. However, this finding is inconsistent with the regulatory mechanism of biofilm formation proposed in Figure 7. Our results in the four selected *S. aureus* strains support the model provided in Figure 7 and do not support the activation of *icaA* expression by SarA. In a previous study [69], quinic acid inhibited the biofilm formation of *S*. *aureus* in a concentration-dependent manner, with increased expression of *agrA*, *icaA*, and *sigB*, consistent with our results.

Furthermore, diosgenin-induced changes in the expression levels of *codY* [34], *mgrA* [34], *saeS* [34], and *saeR* [34], which are known regulators of biofilm formation, were evaluated (Supplementary Figure S2). However, no synchronized gene expression changes occurred with the strain-specific contrasting changes in biofilm formation induced by diosgenin. This suggests that even if these are regulators of biofilm formation, they may not be involved in diosgenin-induced signaling or may not be regulated by changes in gene expression.

In addition to the regulatory model of biofilm formation presented in Figure 7, we observed opposite changes in *sigB* expression in response to diosgenin treatment in a strain-specific manner. Comparing the amino acid sequences of σ^B^ and its upstream regulator, RsbU, from *S*. *aureus* ATCC 6538 and *S*. *aureus* ATCC 29213 strains, we observed differences in amino acids at specific positions. These differences may be responsible for the strain-specific opposite response to the diosgenin treatment. If these amino acid differences are unrelated to strain-specific diosgenin responses, it could be suggested that signal transduction upstream of RsbU confers the strain-specific opposite signals. Further studies are warranted to understand how the same diosgenin treatment can induce opposite changes in *sigB* expression. σ^B^, an alternative general stress response sigma factor, helps *S*. *aureus* to survive under hostile environmental conditions. Therefore, studying the mechanisms of strain-specific opposite changes in *sigB* expression induced by diosgenin may provide insights into the survival strategies of *S*. *aureus* under unfavorable environmental conditions. These further studies will provide clues that can help to prevent and treat various diseases caused by *S*. *aureus*.

## 4. Conclusions

By tracing the intracellular signaling by diosgenin without introducing genetic modifications, this study proposed an integrated model for the regulatory mechanisms of biofilm formation. The existence of strain-specific opposing responses to diosgenin suggests a diversity of signal transduction mechanisms for the regulation of the σ^B^ activity in *S*. *aureus*. Strain-specific responses to the regulation of biofilm formation confer strain-specific selectivity, whereby the subspecies population distribution of *S*. *aureus* may vary depending on the stress conditions imposed. This suggests that it may affect the effectiveness of infection treatments or hygiene processes.

## Data Availability

The data presented in this study are available on reasonable request from the corresponding author.

## Supplementary Materials

The following supporting information can be downloaded at: https://www.mdpi.com/article/10.3390/microorganisms11102376/s1, Figure S1: Effect of diosgenin on cell growth; Figure S2: Diosgenin-induced changes in *codY*, *mgrA*, *saeS*, and *saeR* expression.

## Abbreviations

CFU	Colony-forming units
EPS	Extracellular polymeric substances
MIC	Minimal inhibition concentration
MRSA	Methicillin-resistant *S*. *aureus*
PBS	Phosphate-buffered saline
PIA	Polysaccharide intercellular adhesion
PVC	Polyvinyl chloride
RT-PCR	Real-time polymerase chain reaction
SRAs	Surface-related adhesins
TSA	Tryptic soy agar
TSB	Tryptic soy broth
