# Supplementary material for: Regulation of σB-Dependent Biofilm Formation in Staphylococcus aureus through Strain-Specific Signaling Induced by Diosgenin
Source: Microorganisms. 2023 Sep 23;11(10):2376. doi: 10.3390/microorganisms11102376 (PMC10609180; doi:10.3390/microorganisms11102376)
Supplement: Supplementary file 1 [file microorganisms-11-02376-s001.zip › microorganisms-2572705-supplementary.pdf]

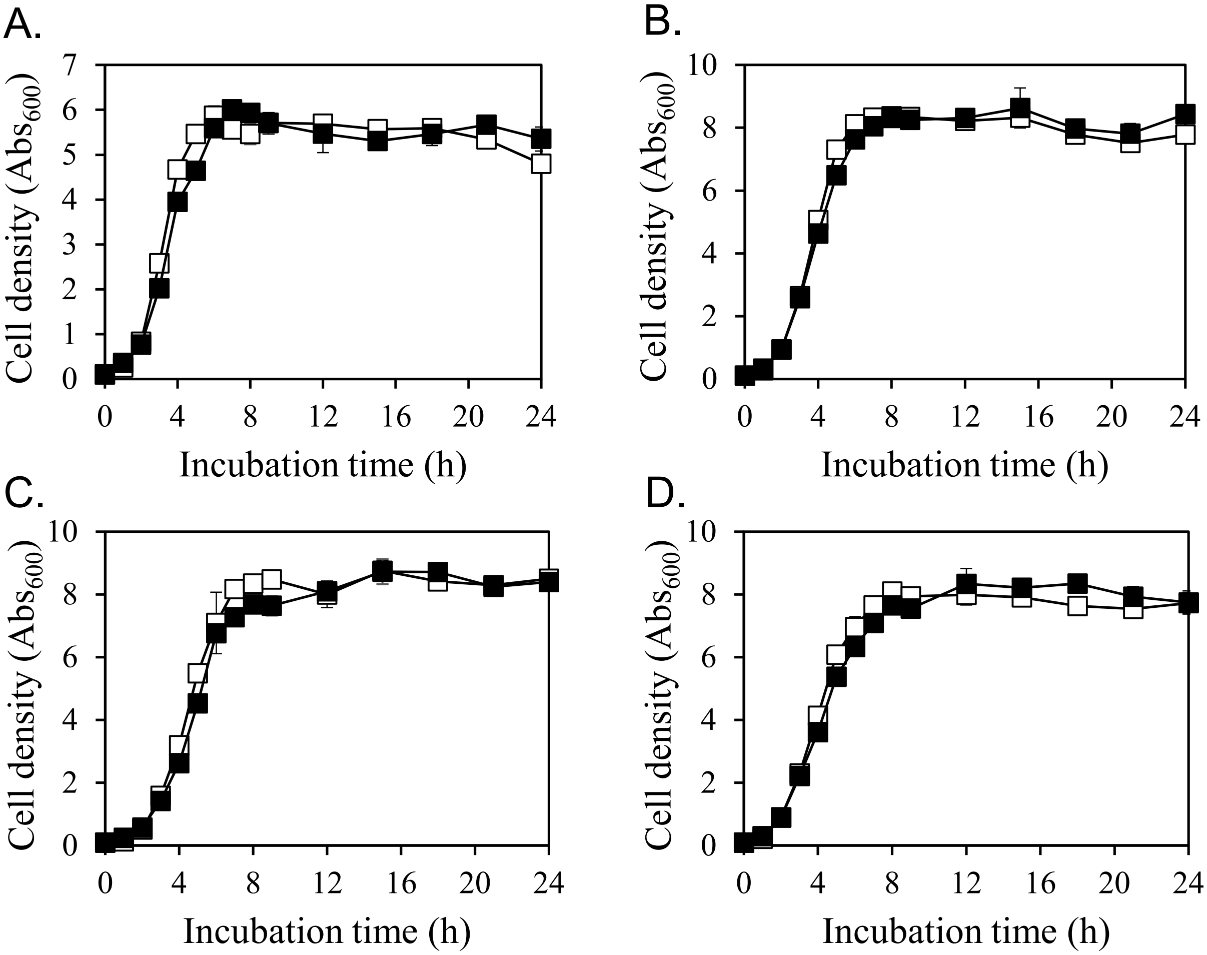

Supplementary Figure S1. Effect of diosgenin on cell growth. The cell growth of *S. aureus* ATCC 6538 (A), *S. aureus* ATCC 29213 (B), MRSA CCARM 3090 (C), and MRSA CCARM 3820 (D) was measured without diosgenin (□) as a control or with 80 μM diosgenin (■). Values were calculated from five independent results, and their standard deviations were shown.

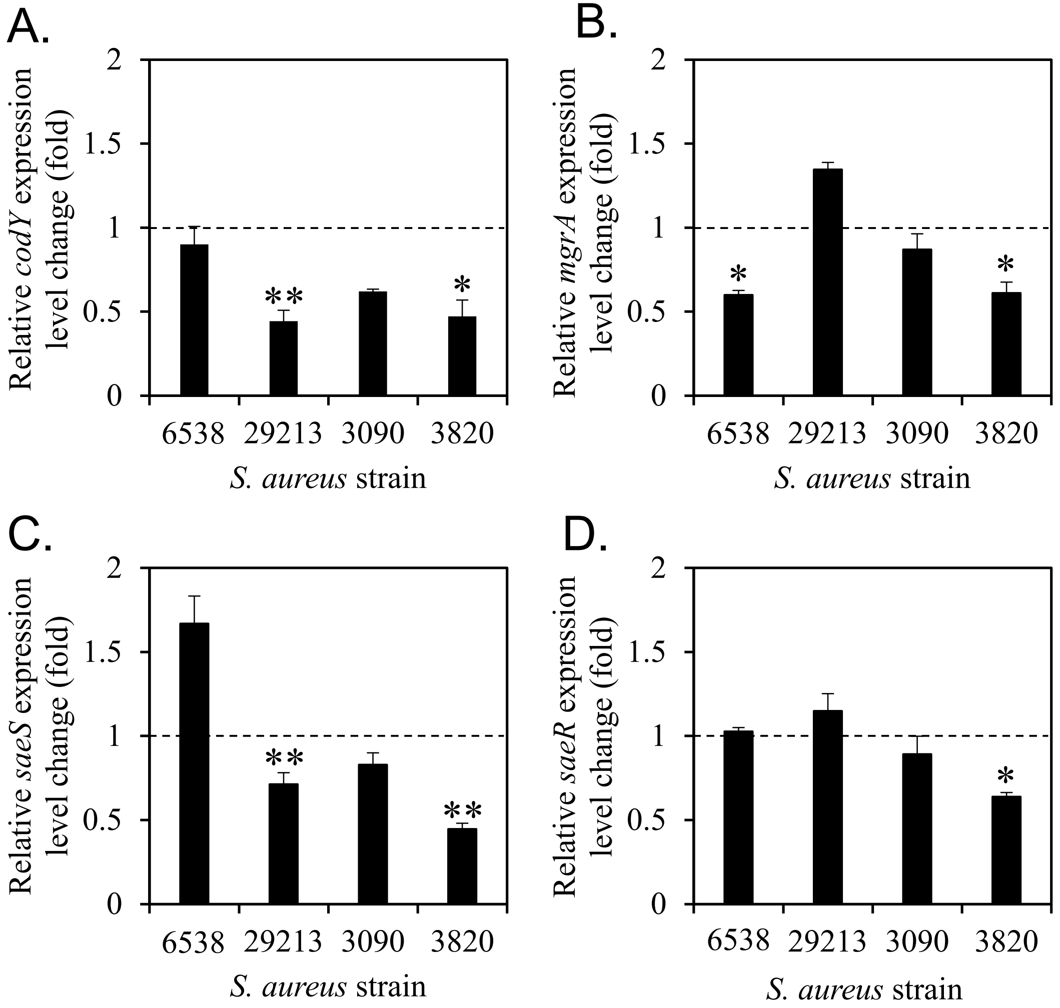

Supplementary Figure S2. Diosgenin-induced changes in *codY*, *mgrA*, *saeS*, and *saeR* expression. *Staphylococcus aureus* ATCC 6538, *S. aureus* ATCC 29213, MRSA CCARM 3090, and MRSA CCARM 3820 was cultured without diosgenin as a control and with 80 mM diosgenin. The primer sequences for amplification were 5'-AAAGAAGCGCGCGATAAAGC-3' and 5'-CTTCCGTACCGCCAAGTTCT-3' for *codY*, 5'-TCACGTTGATCGACTTCGGA-3' and 5'-TGGGATGAATCTCCTGTAAACGT-3' for *mgrA*, 5'-TGTTGCGCGAGTTCATTAGC-3' and 5'-AGCGATGAAGGTATTGGCATT-3' for *saeS*, and 5'-CGCATAGGGACTTCGTGACC-3' and 5'-TCCAAGGGAACTCGTTTTACGT-3' for *saeR*. The experiment was performed according to the section of '2.5. Analysis of Gene Expression Levels Using Real-Time Polymerase Chain Reaction (RT-PCR)' in the manuscript. Values were calculated from three independent results, and their standard deviations are shown. Values that differ from the control with 95% and 99% confidence levels are marked with one and two asterisks, respectively, on top of the bars.
